# Advanced Bile Duct Cancers: A Focused Review on Current and Emerging Systemic Treatments

**DOI:** 10.3390/cancers14071800

**Published:** 2022-04-01

**Authors:** Darren Cowzer, James J. Harding

**Affiliations:** Department of Medicine, Memorial Sloan Kettering Cancer Center, Weill Cornell Medical College, New York, NY 10065, USA; cowzerd@mskcc.org

**Keywords:** cholangiocarcinoma, gallbladder cancer, systemic therapy, immunotherapy, precision medicine

## Abstract

**Simple Summary:**

Biliary tract cancer (BTC), comprising intrahepatic and extrahepatic cholangiocarcinoma as well as gallbladder carcinoma, continues to have poor outcomes for those with unresectable or metastatic disease. The mainstay of therapy has been cytotoxic chemotherapy. A paradigm shift is now occurring due to the widespread availability of next-generation sequencing identifying actionable genomic alterations in a patient subset and the observation that BTC may be sensitive to immune attack. The development of effective targeted therapies is helping to transforming care and combination chemoimmunotherapy appears to improve clinical outcomes. In this review, we discuss the current treatment landscape, available options, and future direction of the treatment of advanced BTC.

**Abstract:**

Cancers arising in the biliary tract are rare, with varied incidence depending on geographical location. As clinical presentation is typically vague with non-specific symptoms, a large proportion of patients present with unresectable or metastatic disease at diagnosis. When unresectable, the mainstay of treatment is cytotoxic chemotherapy; however, despite this, 5-year overall survival remains incredibly poor. Diagnostic molecular pathology, using next-generation sequencing, has identified a high prevalence of targetable alterations in bile duct cancers, which is transforming care. Substantial genomic heterogeneity has been identified depending on both the anatomical location and etiology of disease, with certain alterations enriched for subtypes. In addition, immune checkpoint inhibitors with anti-PD-1/PD-L1 antibodies in combination with chemotherapy are now poised to become the standard first-line treatment option in this disease. Here, we describe the established role of cytotoxic chemotherapy, targeted precision treatments and immunotherapy in what is a rapidly evolving treatment paradigm for advanced biliary tract cancer.

## 1. Introduction

Biliary tract cancers (BTC) account for approximately 3% of all gastrointestinal malignancies and are comprised of intrahepatic cholangiocarcinoma (iCCA), extrahepatic cholangiocarcinoma (eCCA), and gallbladder carcinoma (GBC) [1]. Although classified under the umbrella of BTC based upon shared anatomic and pathohistological characteristics, these tumors exhibit differential epidemiology, biologic behavior, and clinical outcomes [2,3]. Given the low incidence, the lack of validated screening, and the aggressive biology, over 70% of BTC cases are unresectable with clear margins or metastatic at the time of diagnosis [4,5,6]. For such patients, the outlook is dismal with a 5-year survival of 2% [7]. For localized disease, surgical resection is a curative option, but recurrence rates are high and the overall survival (OS) in this setting still remains poor [8,9].

**Figure 1:**
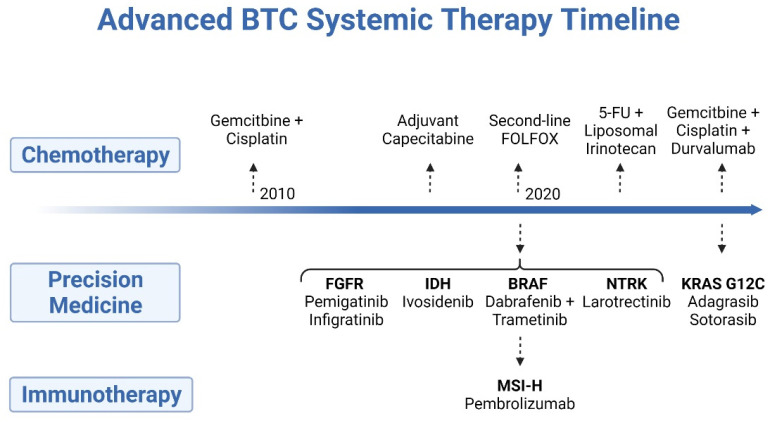
Timeline of systemic treatments developed for the treatment of advanced biliary tract cancer.

Cytotoxic chemotherapy has been the mainstay of systemic treatment and is well established as a means to prolong life and to palliate cancer-related symptoms, though an explosion of drug development in recent years has led to the adoption of several new forms of therapy (Figure 1). Recently, basic and correlative science has revealed that a subset of BTC may elicit an immune response, and prospective studies indicate a role for immune checkpoint inhibition as a potential treatment in this disease [10]. In addition, a wealth of studies have employed whole-genome and -exome next-generation sequencing, which has increased the knowledge of the genomic alterations and molecular phenotype associated with BTC, leading to the successful application of precision medicine in this disease [11,12,13]. Herein, we will review the current state-of-the-art treatment for advanced BTC, with a critical focus on how both new and old systemic treatments will be employed in this disease to improve outcomes. 

## 2. Cytotoxic Chemotherapy

The landmark ABC-02 study established the standard-of-care first-line treatment for the last decade. This phase III study randomized treatment-naïve advanced BTC patients to the combination of gemcitabine and cisplatin versus gemcitabine alone for a maximum of 6 months [14]. The addition of cisplatin to gemcitabine improved median progression-free survival (mPFS) from 5 months to 8 months and median overall survival (mOS) from 8.1 months to 11.7 months (HR 0.64; 95% CI, 0.52 to 0.80; *p* < 0.001). The BT22, a randomized phase II trial of similar design conducted in Japan, supported these finding in a non-Western population and a follow-up study explored the alternative regimen of gemcitabine with S-1, a fluoropyrimidine analogue [15]. This phase III study with 354 patients demonstrated non-inferiority for the non-platinum-based approach. The mOS was 13.4 months in those treated with gemcitabine/cisplatin and 15.1 months in patients treated with gemcitabine/S-1 (HR 0.945; 90% CI, 0.78–1.15; *p* = 0.046 for non-inferiority) [16].

Until recently, there has been a lack of high-level evidence for the benefits of second-line chemotherapy. The ABC-06 study comparing 5-FU and oxaliplatin (FOLFOX) with active symptom control (ASC) (which included the early identification and treatment of biliary complications, e.g., obstruction/sepsis and symptom management) in the second-line setting was the first to demonstrate a survival advantage for chemotherapy [17]. The addition of FOLFOX to ASC improved mOS modestly from 5.3 months to 6.2 months (HR 0.69; 95% CI 0.50–0.97; *p* = 0.031). More recently, liposomal irinotecan in combination with 5-FU was assessed in the phase II NIFTY trial. Following progression on gemcitabine/cisplatin, patients were randomized to either liposomal irinotecan/5-FU or 5-FU alone. Overall response rates (ORR) were higher in the arm containing liposomal irinotecan (15% vs. 6%) as was PFS, the primary endpoint of the study (7.1 vs. 1.4 months; HR 0.56, 95% CI 0.39–0.81; *p* = 0.0019) [18]. 

Taken together, these studies indicate a clear role for combination chemotherapy in both first- and second-line settings in an unselected patient population. Outcomes remain suboptimal, thus clinical research has focused on augmenting results by adding rational agents to a cytotoxic backbone, including more chemotherapy, targeted therapy, and most recently immune checkpoint inhibitors. Regarding the latter, increasing evidence indicates that cytotoxic treatment might induce immunogenic cell death and interfere with mechanism of immune evasion, making it a sensible approach [19]. 

### 2.1. Doublet vs. Triplet Chemotherapy

Treatment intensification with additional cytotoxic chemotherapy regimens has shown benefit in other gastrointestinal malignancies such as colon and pancreas cancer; therefore, there is compelling clinical rationale to test it in BTC [20,21]. The combination of cisplatin, gemcitabine and nab-paclitaxel has shown promising preliminary results [22]. In a phase II study, the triplet combination demonstrated an impressive ORR of 45% and a mPFS of 11.8 months (95% CI, 6 to 15.6) and mOS of 19.2 months (95% CI, 13.2 months to not estimable). Prominent toxicity was noted, however, with 16% of patients withdrawing due to treatment-associated adverse events and ≥grade 3 events in 58% of patients. Treatment efficacy did not appear to be significantly dose dependent and that tolerability could be improved with a reduction in dose. The Southwest Oncology Group (SWOG) is now investigating this triplet combination versus gemcitabine/cisplatin in a phase III trial (NCT03768414). 

Another triplet chemotherapy regimen tested is the combination of 5-FU, irinotecan and oxaliplatin (FOLFIRINOX) with important negative results. In a phase II/III French study, mFOLFIRINOX was compared with gemcitabine/cisplatin in patients with locally advanced unresectable or metastatic BTC, again with treatment continued for a maximum of six months, similar to the ABC-02 study [23]. A total of 191 patients were randomized with a reported mPFS of 6.2 months (95% CI, 5.5 to 7.8) in the mFOLFIRINOX arm and 7.4 months (95% CI, 5.6 to 8.7) in the gemcitabine/cisplatin arm. Median OS was 11.7 months (95% CI, 9.5 to 14.2) in the mFOLFIRINOX arm and 13.8 months (95% CI, 10.9 to 16.1) in those treated with the doublet. There was also importantly no statistical difference in ORR between the two groups. 

A modest survival advantage has been demonstrated in a phase III Japanese study of cisplatin, gemcitabine plus S-1 [24,25]. When compared to gemcitabine/cisplatin, the triplet regimen improved the mPFS by 1.9 months (5.5 months versus 7.4 months, HR 0.75, 95% CI; 0.58–0.97; *p* = 0.0015) and mOS by 0.9 months (12.6 months vs. 13.5 months, HR 0.79. 95% CI, 0.60–1.04; *p* = 0.046). The overall response rate was 41.5% in the three-drug arm compared to 15% in those treated with gemcitabine/cisplatin with reported toxicity similar except for adverse events attributed solely to S1. 

These emerging data indicate that triplet-based regimens may offer a higher objective response rate albeit with a greater risk of toxicity. The effect on survival is modest with the addition of S-1 to cisplatin and gemcitabine and notably FOLFIRINOX maybe harmful compared to traditional doublets in the first-line setting. Awaited confirmatory phase III data from the SWOG-1815 study will further inform doublet vs. triplet chemotherapy in patients with advanced disease (NCT03768414).

### 2.2. ‘Smart’ Chemotherapy

Antibody–drug conjugates (ADCs) have gained much interest in recent years due to their ability to deliver high doses of cytotoxic chemotherapy directly to cancer cells with minimal off-target adverse effects. Targeting HER2 in BTC with ADCs has shown clinical benefit in early-phase studies with other trials ongoing (NCT03821233) and studies are now evaluating targets known to be overexpressed in BTC such as claudin, p-cadherin, and other cell surface molecules [26,27,28] (NCT04683939).

## 3. Immunotherapy

### 3.1. Biomarkers and Patient Selection

Clinical and translational data suggest that a subset of BTC might evade the immune response and several studies have evaluated immune checkpoint blockade [10]. To date, except for microsatellite instability-high (MSI-H) tumors, no clinically meaningful biomarker has been identified though several have been proposed and are being evaluated. 

At a level of the gene, less than 2% of patients with BTC have DNA mismatch repair protein deficiency (dMMR) and/or MSI-high tumors [29]. Multiple studies in solid tumors including BTC have demonstrated durable responses to immunotherapy in MSI-high patients leading to the tumor agnostic approval of immunotherapy in this setting. KEYNOTE-158, a phase II study of pembrolizumab in patients with advanced, pre-treated, non-colorectal MSI/dMMR tumors included 22 patients with BTC, 2 had a complete response and 7 a partial response (ORR 40.9%, Table 1). Median PFS was 4.2 months (95% CI: 2.1-not reached) and mOS of 24.3 months (95% CI: 6.5-not reached) [30]. Thus, it is critical to identify this rare population and treat accordingly. 

Despite approval for use in patients with >10 mut/mb, much debate exists around tumor mutational burden (TMB) and its use as a predictor of response to immunotherapy. Genomic assessment of 412 BTC revealed that 11% had germline mutations in genes associated with the development of a high tumor mutational burden (RAD51D, MLH1, MSH2, POLD1, POLE and ATM), making it an attractive biomarker to assess [31]. In other disease subtypes, however, when MSI-high is excluded from the analysis of patients with high-TMB, its predictive value appears to be limited [32]. 

Wide variation in reports suggests that anywhere between 9.1 and 72.2% of BTCs are PD-L1 positive and like other cancers, it appears that PD-L1 expression in BTC is an imperfect biomarker to predict response to immunotherapy [33,34,35]. Pretreatment PD-L1 expression in most studies discussed below has not shown utility in selecting those who are more likely to respond. However, on-treatment biopsies demonstrating an increase in the level of PD-L1 expression correlated with an improvement in mPFS [36]. PD-L1 did appear to predict improved efficacy in patients treated with a combination TKI and anti-PD-1 therapy [37].

### 3.2. Single-Agent Immune Checkpoint Inhibitors

In the clinic, several single-arm, phase I/II studies have assessed anti-PD-1/PD-L1 therapy (Table 1); however, these studies indicate a low level of antitumor activity to this class of agent in unselected patient populations. Within the Keynote-158 study, a basket study which included a molecularly unselected group of patients with BTC, amongst 104 patients with microsatellite stable BTC, the ORR was 5.8% with a mPFS and mOS of just 2.0 and 7.4 months, respectively [38]. The responses appeared durable with half of patients who responded still with disease control beyond 2 years. A similar phase II study of the anti-PD1 monoclonal antibody nivolumab in heavily pretreated microsatellite stable BTC, the ORR by central review was 11% with a mPFS of 3.68 months and mOS of 14.2 months [39]. Other studies of single-agent immune checkpoint inhibition have yielded similar results, with ORR ranging from 3% to 13% and OS ranging from 5.2 to 8.1 months [38,40,41]. 

**Table 1:** Immune checkpoint Inhibitors in biliary tract cancer.

Immune Checkpoint Inhibitors in Biliary Tract Cancer								
Author	Therapy	Phase	Patient Population	N	Target	ORR (%)	PFS (Months)	OS (Months)
Marabelle et al. *J. Clin. Oncol.* 2020 [30]	Pembrolizumab	II	Chemotherapy refractory Microsatellite instability-high (MSI-H) solid tumors	22	Anti-PD1	40.9	4.2	24.3
Ueno et al. *ESMO* 2018 [42]	Pembrolizumab	II	Chemotherapy refractory Microsatellite stable solid tumors	104	Anti-PD1	5.8	2	7.4
Kim et al. *JAMA Oncol.* 2020 [39]	Nivolumab	II	Chemotherapy refractory biliary tract cancer	54	Anti-PD1	22	3.68	14.2
Ioka et al. *ASCO GI* 2019 [40]	Durvalumab	I	Chemotherapy refractory biliary tract cancer	42	Anti-PD-L1	5	-	8.1
Klein et al. *JAMA Oncol.* 2020 [43]	Nivolumab + ipilimumab	II	Chemotherapy refractory biliary tract cancer	39	Anti-PD1 + anti-CTLA4	23	2.9	5.7
Ioka et al. *ASCO GI* 2019 [40]	Durvalumab + tremelimumab	I	Chemotherapy refractory biliary tract cancer	62	Anti-PD-L1 + anti-CTLA4	10.8	-	10.1
Oh et al. *ASCO* 2020 [36]	Gemcitabine/cisplatin + durvalumab	II	First-line treatment of biliary tract cancer	45	Chemotherapy Anti-PD-L1	73.4	11	18.1
Yoo et al. *ESMO* 2020 [44,45]	Bintrafusp alpha	I *	Chemotherapy refractory biliary tract cancer	30	Bifunctional fusion protein against PD-L1 and TGF-BRII	23.3	-	12.7

ORR = overall response rate, PFS = progression-free survival, and OS = overall survival. * Follow-up phase II/III first-line study INTR@PID BTC 055 discontinued early by sponsor citing it is not likely to meet primary endpoint of OS (NCT04066491).

### 3.3. Combination and Novel Immunotherapy Strategies

The strategy of adding anti-CTLA4-based treatment to monotherapy to improve response has also been explored in early-phase studies. The combination of nivolumab and ipilimumab was tested in a phase II basket study which included patients with advanced BTC (Table 2) [43]. The ORR was 23% and the disease control rate (DCR) was 44%, with responses limited to iCCA and GBC. Despite these response rates and acknowledging the hazards of cross trial comparisons, PFS and OS were not dissimilar to single-agent immune checkpoint inhibition at 2.9 months and 5.7 months, respectively. A similar combination of durvalumab and tremelimumab demonstrated an ORR of just 7.1% with a mOS of 10.1 months [40]. 

Despite a lack of benefit for VEGF inhibition in combination with chemotherapy or single-agent tyrosine kinase inhibitors, there is strong preclinical evidence for their use in combination with immune checkpoint inhibitors and benefits have already been demonstrated in other cancer types [46,47,48]. The inhibition of VEGF promotes T-cell infiltration into tumors and reduces the amount of immunosuppressive regulatory T cells [49]. Thirty-two patients with BTC were treated with the combination of lenvatinib and pembrolizumab following progression on standard chemotherapy with a reported mPFS of 4.9 months and mOS of 11.0 months [37]. Based on these results, the National Comprehensive Cancer Network (NCCN) have listed the combination as a class 2b recommendation for select patient populations and large studies are ongoing to define the operating characteristics of such combinations in BTC (NCT04550624, NCT03895970).

A novel bifunctional fusion protein targeting PD-L1 and transforming growth factor beta (TGF-β), bintrafusp alpha (M7824) was tested in advanced BTC as monotherapy in the second-line setting. Long-term follow up of the phase I iteration of the study reported a mOS of 12.7 months with a 24 month OS rate of 27.7% [44]. Following on from this, a phase II/III first-line study, INTR@PID BTC 055, in combination with chemotherapy was opened (NCT04066491). Just recently, however, the study sponsor has decided to discontinue the study, citing that the trial is not likely to meet its primary endpoint of improving OS [45].

Combinations of immune checkpoint inhibitors with chemotherapy have demonstrated higher response rates where single-agent immunotherapy has been disappointing [50,51]. Promising results have been reported in a phase II setting of BTC, where, in combination with first-line gemcitabine/cisplatin chemotherapy, the addition of durvalumab led to an ORR of 73.3% with a 100% DCR. Median PFS was 11 months and mOS was 18.1 months [36]. Following on from this, the phase III TOPAZ-1 study was developed (NCT03875235), with results recently reported at GI ASCO 2022 [41,52,53]. This double-blind placebo-controlled trial randomized previously untreated unresectable locally advanced or metastatic BTC patients 1:1 to receive either durvalumab or placebo in combination with gemcitabine/cisplatin. Chemotherapy was continued for a maximum of 8 cycles with durvalumab maintenance ongoing until progression or unacceptable toxicity. The primary objective was overall survival. Both OS (HR, 0.80; 95% CI, 0.66–0.97; *p* = 0.021) and PFS (HR, 0.75; 95% CI, 0.64–0.89; *p* = 0.001) were improved with the addition of durvalumab when compared to placebo. While the full results are awaited, this is likely to result in a change in the standard-of-care first-line treatment for patients with advanced BTC.

## 4. Molecular Targets within Biliary Tract Cancer

**Figure 2:**
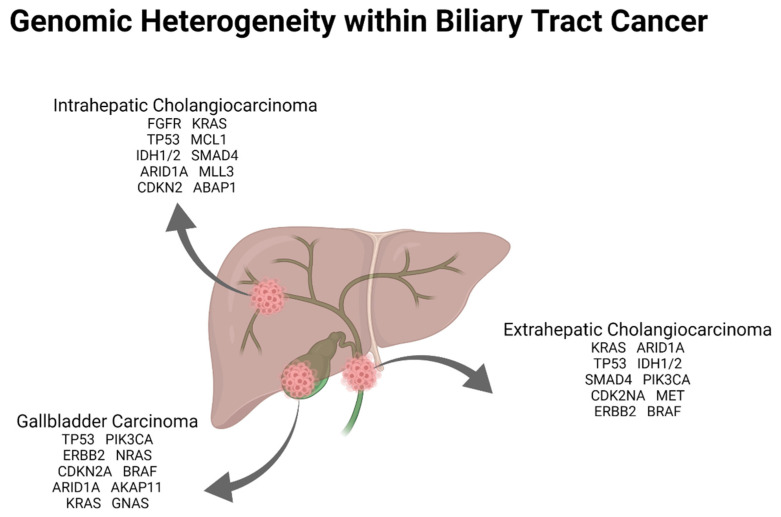
Most common genomic alterations identified based on anatomic location of biliary tract cancer. Frequency of alterations listed is based on Nakamura et al. *Nat. Genet.* 2015 [10], Lowery et al. *Clin. Cancer Res.* 2018 [12], Javle et al. *Cancer* 2016 [13] and Wardell et al. *J. Hepatol.* 2018 [11].

Molecular characterization of BTC has shown actionable targets ranging from 25 to 55% reported in different studies depending largely on the location of the primary and the tissue that is tested (Figure 2) [10,12]. Several key genomic alterations have been identified in BTC with targeted precision therapies subsequently yielding encouraging results (Table 2). 

**Table 2:** Targeted therapy in biliary tract cancer.

Targeted Therapy in Biliary Tract Cancer								
Author	Therapy	Phase	Patient Population	N	Target	ORR (%)	PFS (Months)	OS (Months)
Abou-Alfa et al. *Lancet Oncol.* 2020 [54]	Pemigatinib	II	Chemotherapy refractory patients with FGFR2 fusion/rearrangements in iCCA	107	FGFR1-3	35.5	6.9	21.1
Javle et al. *Lancet Gastro.* 2021 [55]	Infigratinib	II	Chemotherapy refractory patients with FGFR fusion/rearrangements in iCCA	108	FGFR1-4	23.1	7.3	-
Bridgewater et al. *ESMO* 2020 [56,57]	Futibatinib	II	Chemotherapy refractory patients (inc those prior FGFR treatment) with FGFR fusion/rearrangements in iCCA	67	FGFR1-4	37.3	7.2	-
Mazzaferro et al. *Br. J. Cancer* 2019 [58]	Derazantinib	I/II	Chemotherapy refractory patients with FGFR fusion/rearrangements in iCCA	29	Non-selective multikinase inhibitor	20.7	5.7	-
Park et al. *ASCO GI* 2019 [59]	Erdafitinib	II	Chemotherapy refractory patients with FGFR fusion/rearrangements in iCCA	12	FGFR1-4	50	5.6	-
Ng et al. *Hepatobiliary Surg.* 2019 [60]	Debio 1347	I	Chemotherapy refractory patients with FGFR fusion/rearrangements in solid tumors	9	FGFR1-3	22	-	-
Abou-Alfa et al. *Lancet Oncol.* 2020 [54]	Ivosidenib Placebo	III	Chemotherapy refractory patients with iCCA	185	IDH1	2	2.7 1.4 *p* < 0.001	10.8 9.7 *p* = 0.06
Subbiah et al. *Lancet Oncol.* 2020 [61]	Dabrafenib + Trametinib	II	Refractory solid tumors	33	BRAF + MEK	51	7.2	11.3
Demols et al. *Ann. Oncol.* 2020 [62]	Regorafenib vs. Placebo	II	Refractory BTC	66	VEGF	0	3 1.5 *p* = 0.004	5.3 5.1 *p* = 0.21
Javle et al. *Lancet Oncol.* 2021 [63]	Trastuzumab + Pertuzumab	II	Refractory solid tumors including BTC	39	HER2 amplification or mutation	23%	4	10.9

### 4.1. IDH

Isocitrate dehydrogenase (IDH) 1/2 gain-of-function mutations result in changes in cellular metabolism and the ultimate accumulation of 2-hydroyglutarate (2-HG) plays a key role in interfering with cellular differentiation and therefore promoting tumorigenesis [64]. Of the three isoforms of IDH, IDH1/2 appear to have the most relevance to cancer and have therefore been the focus of drug development. The most common mutations are typically point mutations occurring the arginine residue [54] in the catalytic pockets: IDH1 (codon R132, more specifically R132C, R123G) and IDH2 (codon R172 or 140).

Ivosidenib (AG-120) is the first oral targeted inhibitor of IDH1 that has demonstrated benefit in pretreated IDH1-mutant advanced cholangiocarcinoma patients. Within the ClarIDHy study, R132C was the most common IDH1 mutation identified (70%) followed by R132L (17%) with 90% of occurring in iCCA [54]. When compared to placebo, PFS in the ivosidenib group was improved by 1.3 months from 1.4 to 2.7 months (HR 0.37; 95% CI 0.25–0.54; *p* < 0.0001). In those treated with ivosidenib 6 month PFS was 32% compared to 0% in those treated with placebo. There was, however, no statistical difference in OS in the intention to treat (ITT) population likely related to crossover which occurred at the time of radiological progression in more than half of those who received placebo (10.3 vs. 7.5 months, HR, 0.79, 95% CI, 0.56–1.12; *p*  = 0 .09). When survival was adjusted for crossover, the OS in the placebo group was just 5.1 months [65].

Other IDH1 and IDH2 inhibitors, alone or combination, are currently under investigation in clinical trials. LY3410738, a next-generation, selective covalent IDH1/2 inhibitor, was designed to break established resistance mechanism observed with ivosidenib (NCT04521686) [66,67,68]. IDH alterations also appear to promote a BRCA-like phenotype and preclinical data have suggested synergy with poly (ADP-ribose) polymerase inhibitors (PARPi) [69]. Early-phase studies are underway examining the combination IDH inhibition plus immune checkpoint blockade (NCT04056910) and PARPi in combination with immunotherapy (NCT04306367, NCT03991832). Indeed, IDH1 inhibition appears to alter the immune microenvironment and 2HG has inhibitor effects on cytotoxic T cells, supporting IO/IDH combinations. 

### 4.2. FGFR

The fibroblast growth factor (FGFR) pathway consists of four conserved transmembrane receptors with intracellular kinase domains. FGFR activation and signaling are dependent on ligand-induced dimerization, which leads to intracellular phosphorylation, signal transduction and ultimately gene transcription [70]. A number of different genomic alterations have been identified in FGFR that lead to aberrant signal transduction, with a preponderance of rearrangements or fusions over amplifications and mutations [71]. FGFR2 fusions appear to be the most common alteration identified in iCCA, with the most common fusion partner being BICC1 [72,73,74]. Downstream pathways influenced by constitutive activation of the FGFR pathway include the Ras–Raf–MEK–ERK and the PI3K–AKT–mTOR pathways, both critical to tumorigenesis [72,73,74]. 

Pemigatinib is the first FGFR inhibitor that has been approved for use by the Food and Drug Administration (FDA) for use in patients with iCCA that harbor FGFR2 fusions or gene rearrangements. This oral inhibitor of FGFR1-3 demonstrated activity in the multicenter phase II FIGHT-202 study. Pretreated BTC patients were treated with 13.5 mg of pemigatinib once daily (21-day cycle; 2 weeks on 1 week off) and 147 patients were enrolled in the study: 107 with FGFR2 fusions/rearrangements, 20 with other FGFR alterations and 18 with no alteration in FGFR. Patients with FGFR fusions or rearrangements had an objective response rate of 35.5% and treatment was reported as tolerable. 

Infigratinib, another FGFR1-3 inhibitor, demonstrated an ORR of 30.1% in a pretreated cholangiocarcinoma population with FGFR2 fusions/translocations. Median PFS in this study was 6.8 months with a mOS of 12.5 months [55]. Both pemigatinib and infigratinib are now under investigation in randomized phase III studies in comparison with gemcitabine/cisplatin in the first-line setting for those with FGFR gene fusions/rearrangements (NCT03656536, NCT03773302).

Several other FGFR inhibitors are in clinical development [58,75,76,77]. Beyond FGFR fusions, futibantinb (TAS-120), a covalent inhibitor, exhibits biochemical properties, allowing it to break established acquired resistance mechanisms with an ORR of 30.8% [55,56,58,72,73,74,75,76,77,78]. Efficacy in the first-line setting compared with standard chemotherapy in those with FGFR rearrangements is currently under evaluation (NCT04093362). 

Innate primary resistance to therapy remains poorly understood, with suggestions that certain co-occurring genetic alterations determine resistance to FGFR inhibition. Despite overall positive results and response rates, secondary resistance to therapy appears to be ubiquitous—mainly in the form of point mutations occurring in the kinase domain of FGFR2 (i.e., gatekeeper mutations such as V565F, I or L and molecular brakes such as E566A, and N550D, H, K, or T) [57,79,80,81]. On-target secondary resistance results in inefficacy of therapy despite continued reliance on FGFR signaling by the cancer. As alluded to earlier, it is possible that further response can be achieved with differing therapies; however, this is not proven clinically as of yet [55,56,58,72,74,75,76,77,78,79,80,81]. Efforts are ongoing to ascertain the most appropriate sequencing and combination of therapies as well as developing new agents capable of overcoming resistance [80].

### 4.3. HER Receptor Targeting

HER2 overexpression or amplification is seen in approximately 10–16% of GBC and approximately 5–11% of eCCA and activating missense mutations have also be described at a low frequency, prompting a number of single-arm studies of anti-HER2 treatment in BTC [82,83]. The MyPathway phase IIa basket study examined the combination dual anti-HER2 antibodies pertuzumab and trastuzumab [63]. Here, the ORR was 22% and patients with GBC patients appeared to have the more favorable outcomes. ZW25, zanidatamab, is a bispecific HER2 antibody that binds two separate epitopes of HER2 and it too has shown favorable results in a basket phase I. This agent is now being investigated in a global single-arm phase II study (NCT03929666). Initial phase I data from a basket study that included two patients with BTC treated with trastuzumab-deruxtecan (TDxD) led to a partial response both patients, prompting several TDxD basket studies in advanced HER2-positive solid tumors [26] (NCT04639219). ZW49, a combination of zanidatamab with a cleavable linker that delivers an auristatin cytotoxin to HER2-positive tumor cells, is also under investigation (NCT03821233). Neratinib, an oral pan-HER inhibitor, has also demonstrated efficacy in heavily pretreated BTC harboring HER2 mutations in the SUMMIT trial [84]. The ORR in 25 patients was 16%, and a similar trend was observed favoring improved outcomes for those with GBC. The data from these studies help support evaluating HER2 in the first-line setting. Trastuzumab is being testing in combination with cisplatin/gemcitabine in the BILHER study (NCT03613168) and it is expected that more combinations of novel agents with both cytotoxic and immune-based treatments will emerge.

### 4.4. BRAF

BRAF mutations are identified in approximately 3–7% of BTC and are enriched in iCCA [85,86,87]. Similar to what is seen in BRAF^V600E^ mutant colorectal cancer, patients with BTC that harbor these mutations are more likely to present with more locally advanced disease and worse overall survival compared to those that are BRAF wild type [88]. Having previously demonstrated positive response rates for single-agent BRAF inhibition in BTC, the basket phase II ROAR trial examined the role of dabrafenib and trametinib in solid tumors harboring BRAF^V600E^ mutations [61,89]. Forty-three patients with BTC, 91% of which had iCCA, were treated with the combination of BRAF and MEK inhibitors. The overall response rate was 51%, mPFS of 9 months and mOS of 14 months, like the results demonstrated with other targeted therapies in BTC. The NCI-MATCH trial subprotocol H also demonstrated partial responses in three of four patients with BRAF^V600E^ mutant BTC treated with dabrafenib and trametinib [90].

### 4.5. Homologous Recombination Deficiency

Genes implicated in DNA damage response (DDR) and homologous recombination including but not limited to BRCA1/2, ATM, BAP1, CHEK1/2, PALB2, ARID1A, ATRX and BARD1 have been identified in up to 20% of BTC, predominately in eCCA [13,91,92]. There is a suggestion, as is the case in other cancers associated with alterations in DDR genes, that these subtypes of BTC may be more responsive to platinum-based therapy [93]. Data examining the outcome of BTC that harbor mutations in DDR pathway genes are in limited retrospective reports [94]. The efficacy of PARPi, which have improved outcomes in BRCA-associated breast cancer and homologous recombination-deficient (HRD) ovarian cancer, in BTC is currently not known, with several studies ongoing (NCT03991832). Given the anatomical and histological similarities with pancreatic ductal adenocarcinoma where PARP inhibition demonstrated an improvement in PFS with the addition of olaparib, their role in BTC is under investigation both as monotherapy and in combination with chemotherapy or immunotherapy (NCT04895046, NCT04042831) [95]. 

### 4.6. Infrequent Alterations (KRAS, G12C and NTRK)

Activating mutations in KRAS occur in between 7 and 40% of BTC depending on the anatomical location of BTC and portend worse outcomes [92,96,97]. Similar to the presence of TP53 and CDK2A, KRAS mutations predict a poor outcome in both resectable and unresectable disease [98]. Targeting of KRAS mutations has been challenging historically and most are not actionable. The only approved therapy for KRAS mutant solid tumors is sotorasib, which specifically targets KRAS^G12C^ mutations, an alteration found in less than 1% of BTCs. Within the phase I study of sotorasib, CodeBreak100, one patient with BTC had stable disease [99]. The phase II KRYSTAL-1 trial of adagrasib in patients with KRAS^G12C^ mutations demonstrated more positive results for those with BTC. Eight patients were treated with a partial response rate of 50% and the disease control rate of 100%. Enrollment for this study continues alongside a early access program for those patients with KRAS^G12C^ mutations [100].

Genomic translocations in the neurotrophic tyrosine receptor kinase (NTRK) that lead to constitutive activation are also rare in cholangiocarcinoma [96]. Both larotrectinib and entrectinib, highly selective inhibitors of tropomyosin receptor kinase, are approved for use by the FDA for use in adults and children in advanced cancer with an NTRK gene fusion. The evidence in BTC, however, comes from the 55 patient basket study that contained 2 patients with BTC, 1 of whom had a partial response to treatment [101]. The entrectinib study enrolled one patient with NTRK-positive cholangiocarcinoma who achieved a partial response [102].

## 5. Conclusions and Future Directions

With minimal progress in over a decade since the reporting of the ABC-02 study, combination chemotherapy plus immunotherapy along with molecular profiling and precision oncology are transforming the treatment paradigm in advanced BTC. In what was once an area of oncology with limited treatment options, collaboration and innovation have led to the approval of multiple therapeutic options. With the wealth of information now available and reduced cost as well as routine access to panel-based genomic assessment, patients are now classified as having a targetable alteration or not ultimately dictating their treatment path. Although single-agent immune checkpoint inhibitors have yet to demonstrate a real clinically meaningful benefit in any cohort outside of the small number with microsatellite unstable disease, it appears that the combination of immunotherapy with chemotherapy does lead to improved outcomes for patients with BTC in the first-line setting and likely represents a paradigm shift in treating the disease.

Several notable limitations are apparent. Despite impressive response rates with targeted treatments in a disease that has only demonstrated improvements in OS with one chemotherapeutic combination, primary resistance is common and secondary resistance is inevitable. The focus now turns to ways to define and overcome resistance with novel agents and rational combination therapy. Further challenges now exist in demonstrating benefits for these agents targeting driver genomic alterations in the first-line setting where improved second-line options are available. Likewise, outside of MSI-high, biomarkers of immune response remain elusive.

A plethora of large-scale randomized studies are underway attempting to answer the questions around biomarkers, drug combinations, and sequence of therapies in patients with BTC. Whether cytotoxic chemotherapy, targeted therapy, or immunotherapy, either alone or some combination, wins out as the preferred first-line option, the optimal sequencing of therapies will likely remain an unanswered question and will require a personalized approach.
